# Seroprevalence, clinical characteristics and risk factors for dengue virus infection among febrile adult patients in a Nigerian tertiary hospital

**DOI:** 10.11604/pamj.2026.53.128.48009

**Published:** 2026-03-13

**Authors:** Juliet Ijeoma Mmerem, Uchechukwu Sonny Unigwe, Mafuka Simon Johnson, Chinedu Michael Chukwubike, Chukwudi Christian Umenzekwe, Michael Onyebuchi Iroezindu

**Affiliations:** 1 Department of Medicine, University of Nigeria Teaching Hospital, Ituku/Ozalla, Enugu State, Enugu, Nigeria,; 2 Department of Internal Medicine, Federal Medical Centre, Owerri, Imo State, Nigeria,; 3 Department of Microbiology, University of Nigeria Teaching Hospital, Ituku/Ozalla, Enugu State, Enugu, Nigeria,; 4 Department of Medicine, Nnamdi Azikwe University Teaching Hospital, Nnewi, Anambra State, Nigeria

**Keywords:** Coinfection, case-control, dengue, fever, malaria, Nigeria, risk factors, seroprevalence

## Abstract

**Introduction:**

dengue is a neglected tropical disease. Underdiagnosis of dengue in the endemic sub-Saharan Africa region has contributed to poor characterisation of disease burden. Here, we determined the seroprevalence, clinical characteristics and risk factors of dengue infection among febrile patients at the Federal Medical Centre, Owerri.

**Methods:**

this case-control study included 90 adult febrile cases and 90 apparently healthy controls, sampled consecutively. Data were obtained using an interviewer-administered questionnaire. Blood was tested for anti-dengue IgG and IgM antibodies, dengue non-structural protein antigen, malaria parasitaemia, full blood count, liver function and kidney function tests. Multivariate analysis determined factors independently associated with dengue seropositivity using a p-value < 0.25 on the Chi-square test. A two-tailed p-value < 0.05 was considered statistically significant.

**Results:**

the participants comprised 48 (53.3%) females in each group with a mean age of 39.3 ± 16.4 years for febrile patients and 38.3 ± 12.5 years for the controls (p=0.642). The seroprevalence of recent dengue virus (DENV) infection (IgM or NS1) was 33.3% in febrile participants vs. 17.8% in controls (p=0.017). For past DENV exposure, IgG only, the difference in seropositivity was also statistically significantly higher in the febrile group compared to control (62.2% vs. 40.0%, p=0.003). Malaria parasitemia was significantly more frequent in dengue IgM seropositive febrile participants compared to the seronegative febrile participants (76.7% vs 55.0%, p=0.046). Only having window /door nets (adjusted odds ratio [AOR]-3.67, 95% CI 1.02 to 8.21, p=0.045) was an independent predictor of recent dengue virus infection, while none of the variables assessed predicted past DENV infection.

**Conclusion:**

this study showed high dengue seroprevalence. Dengue-malaria co-infection highlights the need for clinicians and policymakers to consider laboratory evaluation for dengue in the routine assessment of febrile patients in sub-Saharan Africa.

## Introduction

Dengue is a tropical disease caused by the dengue virus (DENV) and principally transmitted by the *Aedes aegypti* mosquito [1]. It is the most common arthropod-borne viral infection, with over 3.9 billion people at risk worldwide and a projected 6.1 billion to be at risk by 2080 [2,3]. It is a viral haemorrhagic fever that commonly presents asymptomatically [1,4]. According to the World Health Organisation (WHO), DENV infection may result in dengue with or without warning signs, and severe dengue, which may eventually result in death [5]. So far, over 56 million dengue cases have been documented in 102 countries between 1924 and 2023. Local transmission and possible endemicity in Africa have also been reported [3,6,7]. Evidence suggests that dengue remains a global health challenge, as a global rise in incidence, death, and disability-adjusted life-years (DALYs) due to dengue was observed at the country level for the majority of 195 countries and territories [6,8]. Also, increased dengue episodes were observed for most countries and territories following an analysis of the global disease burden (GBD) 2019 [9].

Despite these global estimates, Africa's precise disease burden and distribution remain poorly characterised. Globally, the seroprevalence of DENV infection in the general populace varies from 12.5% to 86.7% [10-13]. The seroprevalence of DENV also differs widely among febrile persons, ranging from 1.8% to 90.0% [14-18]. In Nigeria, as in most sub-Saharan African (SSA) countries, dengue diagnostics are not routinely performed, hence limiting disease knowledge and epidemiology despite DENV being first isolated in Nigeria decades ago [19]. Studies suggest that dengue may account for some undiagnosed febrile complaints in Nigeria [14,20,21]. Acute febrile illnesses are common in the tropics, but incidentally most acute febrile illnesses in the tropics have similar symptomatology of myalgia, headache, sore throat, malaise and anorexia early on in the disease [22]. This makes distinguishing dengue solely on clinical features rather difficult. Also, the SSA climate provides enabling mosquito vector conditions as inadequate drainage systems, poor urban planning, dilapidated roads, and indiscriminate solid waste disposal within settlements promote stagnant water bodies to serve as mosquito breeding sites [23-25]. Being a potentially fatal infectious disease of the tropics [1], dengue lacks sufficient public health attention in SSA as compared to diseases like malaria, tuberculosis, HIV/AIDS, Ebola and Lassa fever. With the increasing frequency of dengue epidemics worldwide, there is a need for a better understanding of the epidemiology of dengue infections in Nigeria [1,6]. Hence, we determined the seroprevalence of DENV infection, described the clinical characteristics of dengue IgM seropositive febrile patients and identified risk factors associated with recent and past dengue infection among febrile adult patients in a Nigerian tertiary care hospital.

## Methods

**Study design, study site, and study population:** this was a case-control study in which cases and controls were matched 1: 1 for sex and age group. Ethical approval for the study was obtained from the Federal Medical Centre (FMC) research ethics committee. Written informed consent was obtained from each participant before recruitment.

The study was initially scheduled to be conducted over six months; however, due to disruptions from the coronavirus disease 2019 (COVID-19) pandemic, the study was conducted over eight months. The study population were persons who presented to the FMC Owerri in South-East Nigeria from 1^st^ February to 30^th^ September 2020. This period cut across the dry and rainy seasons, the two climatic seasons in Nigeria. The FMC Owerri is the largest tertiary hospital in Imo State. She serves as a referral centre for hospitals within the State's geographical region and neighbouring border communities of Abia, Anambra, Enugu and Rivers. Imo State has an estimated population of about 3.9 million [26]. The hospital's general outpatient clinic operates as a primary healthcare point for residents within the State capital. The study population were patients seen at the hospital's general outpatient, medical outpatient, and emergency unit. Eligibility for cases: i) adult patients aged 18 years or more who reside in the geographical region served by FMC Owerri, and ii) presence of acute febrile illness. Eligible controls met the following criteria: i) apparently healthy adults aged 18 years or more who reside in the same geographical area served by FMC; ii) absence of fever in the preceding 4 weeks. Convenience sampling was done with participants recruited consecutively. Pregnant women were excluded.

**Sample size estimation**: using Fischer's formula [27], given a prior prevalence of 4.8% in South-South Nigeria [20], the calculated minimum sample size was 70. Accounting for possible non-response, the sample size was rounded up to 90. Hence, 90 febrile adult patients and 90 age and sex-matched controls were recruited.

**Data collection and outcome measures**: an interviewer-administered questionnaire was developed following a literature review of prior studies on dengue. This captured socio-demographics, clinical symptoms and signs, including those suggestive of viral haemorrhagic fever (VHF), mosquito control practices, dengue transmission risk factors (exposure) and medical comorbidities. Socio-demographic information included age, sex, residence (rural or urban), educational status, marital status, and occupation. Assessment of dengue exposure via mosquito control practices focused on sleeping under insecticide-treated bed nets (ITNs), use of door/window nets, environmental control measures, and use of indoor insecticides. Dengue transmission risk factors assessed included recent blood transfusion, organ transplant, and travel history. The outcome of interest was positivity to DENV markers (IgG, IgM or NS1) while malaria parasitemia was considered a potential confounder, hence assayed for.

**Blood sampling and laboratory analysis**: in total, 14 mL of venous blood was obtained per eligible participant using standard aseptic procedures. Four (4) mL was collected in a clot activator bottle that derived serum for dengue antibodies (IgM and IgG) testing after centrifugation at 1500 revolutions/minute for four minutes. Two (2) mL of venous blood was collected in an ethylene diamine tetraacetic acid (EDTA) bottle for full blood count (FBC). A drop of blood each was used for malaria parasite detection using malaria rapid diagnostic test kit (RDT) SD Bioline (Kyonggi, Korea) and NS1 antigen detection using NS1 RDT. Four (4) mL of blood was collected in a plain bottle for bilirubin assay and liver enzymes (aspartate aminotransferase [AST], alanine aminotransferase [ALT] and alkaline phosphatase [ALP]). Liver enzymes were estimated by colourimetric enzymatic assay. Another 4 mL of blood was collected in a citrate bottle for prothrombin time (PT) and international normalised ratio (INR) using a one-step clotting pathway assay. Pregnancy was excluded in females of reproductive age using the Assured® urine pregnancy test kit.

**Dengue serological testing**: serum preserved temporarily (one week) at a 4°C refrigerator in the FMC laboratory complex was couriered, maintaining cold chain in a triple packaging system to the Virology Laboratory, University of Nigeria Teaching Hospital (UNTH) Enugu. There, it was stored at -80 °C until processed. The serum was tested for the presence of antibodies to DENV by standard ELISA techniques [1]. Demeditec dengue IgM and IgG ELISA kits (DENM0120, Lot: DENM-150, Kiel, Germany and REF: DENG0120, Lot: DENG-151, Kiel, Germany, respectively) were utilised according to the manufacturer's instructions [28,29]. The Ig M kit had a sensitivity of 91.8% and specificity of 96.6% while the IgG kit had a sensitivity of 100.0% and specificity of 98.0% [28,29]. The optical density (OD) result was calculated, and the result was interpreted as positive, negative, or equivocal. Equivocal results were considered negative. NS1 antigen was assayed by NADAL dengue NS1 Ag kit (Ref: 532016n-25, Lot: DEC 20080007, Moers, Germany), a two-step rapid immunochromatographic test read off after 20 minutes. Participants positive for DENV IgG, IgM, and/or NS1 antigen based on the cut-off points of the test kits were considered to be DENV seropositive. Clinical interpretation of dengue serologic results was done using serological markers (IgM, IgG and NS1) in conjunction with duration of fever, as this has been shown to provide good clinical guidance for the provisional diagnosis of dengue [29].

### Definition of terms and laboratory cut-off values in line with the institution's reference range

Acute febrile illness was defined as any illness associated with fever (axillary temperature ≥37.5°C and/or self-report) lasting ≤ 2 weeks [14,30]. Apparently healthy: not currently ill or reporting any acute illness in the preceding four weeks. Recent dengue infection: presence of anti-dengue IgM antibodies or NS1 antigenemia [31]. Past dengue infection: presence of anti-dengue IgG antibodies [31]. Malaria parasitemia (seropositivity) was positive on RDT using the manufacturer's instructions.

Laboratory cut offs: Anaemia: haemoglobin (Hb) < 10g/dl, Leucopenia: total white blood cell count (WBC) < 4,000 cells/L, where neutrophils contribute at least 40-75% and lymphocytes 20-45%, Thrombocytopenia: PLT < 150 x 109/L, Prolonged Prothrombin time: > 14 seconds, Prolonged INR: INR >1.2, Hyperbilirubinemia: > 17 µmoles/L, Elevated liver enzymes: AST > 40IU/L, ALT > 40IU/L, ALP > 150IU/L

**Quality control**: this was maintained in line with local laboratory standard quality control measures and with the aid of the calibrator, a positive and negative control that accompanied the dengue manufacturer's kit [29].

**Data analysis**: data was entered and analysed using the IBM SPSS version 25.0 analytical software. Continuous variables were expressed as mean with standard deviation (SD) when normally distributed or median with interquartile range (IR) when skewed. Categorical variables were expressed as proportions/frequencies. The proportion of those seropositive for DENV serological markers was depicted using the bar chart. Categorical variables were compared using the Chi-square test or Fisher's Exact test as appropriate. For comparison of continuous variables, Student's t-test was used for variables that were normally distributed, and the Mann-Whitney U test (Wilcoxon rank-sum) for skewed distributions. Test of association of dichotomised independent variables (sociodemographic, mosquito transmission and comorbid factors) with outcome of interest; dengue seropositivity was done using the Chi-square test. The likelihood of these factors was determined based on the odds ratios (OR) with its 95% confidence interval (CI). Variables with p-value < 0.25 on bivariate analysis, such as sex (male versus female), malaria parasitaemia (yes or no), and hypertension (yes or no), were selected for regression analysis. Multivariate logistic regression was used to determine predictors of recent and past DENV infection and the adjusted odds ratios (AOR) with 95% CI. Two tailed p-value < 0.05 was considered statistically significant.

## Results

**Epidemiological/demographic characteristics of the patients**: the socio-demographic characteristics of the febrile participants and the control group are summarised in Table 1. In each group, the study participants consisted of 48 (53.3%) females and 42 (46.7%) males in a ratio of 1.1: 1 (p=1.000). The mean age of the febrile participants and the control group was comparable (39.3 ± 16.4 years vs. 38.3 ± 12.5, t = 0.466; p = 0.642). Both study participants, 57 (63.3%) and controls, 64 (71.1%), resided mainly in urban areas. (p=0.266). Both groups were comparable in the educational level attained (p=0.418) but differed in their occupational groups, which was statistically significant (p=0.001).

**Table 1:** socio-demographic characteristics of the study participants

Characteristics	Febrile group N=90, n (%)	Control group N=90, n (%)	Pearsons Chi-square (χ2)	p-value
**Sex**	-	-	-	
Male	42 (46.7)	42 (46.7)	0.000	1.000
Female	48 (53.3)	48 (53.3)	-	-
**Age group (years)**	-	-	-	-
18-30	31 (34.4)	27 (30.0)	1.825	0.610
31-45	36 (40.0)	43 (47.8)	-	-
46-60	12 (13.3)	13 (14.4)	-	-
>60	11 (12.2)	7 (7.8)	-	-
**Residential area**	-	-	-	-
Urban	57 (63.3)	64 (71.1)	1.235	0.266
Rural	33 (36.7)	26 (28.9)	-	-
**Marital status**	-	-	-	-
Single	31 (34.4)	41 (45.6)	5.636	0.131
Married	53 (58.9)	48 (53.3)	-	-
Divorced/separated	3 (3.3)	0 (0.0)	-	-
Widowed	3 (3.3)	1 (1.1)	-	-
**Occupation**	-	-	-	-
Artisan	18 (20.0)	5 (5.6)	38.778	0.001*
Civil servants	28 (31.1)	68 (75.6)	-	-
Trader	25 (27.8)	7 (7.8)	-	-
Unemployed	11 (12.2)	3 (3.3)	-	-
Students	8 (8.9)	7 (7.8)	-	-
**Educational status**	-	-	-	-
None	5 (5.6)	2 (2.2)	2.836	0.418
Primary	9 (10.0)	6 (6.7)	-	-
Secondary	29 (32.2)	26 (28.9)	-	-
Tertiary	47 (52.2)	56 (62.2)	-	-

* Statistically significant at p<0.05

**Figure 1:**
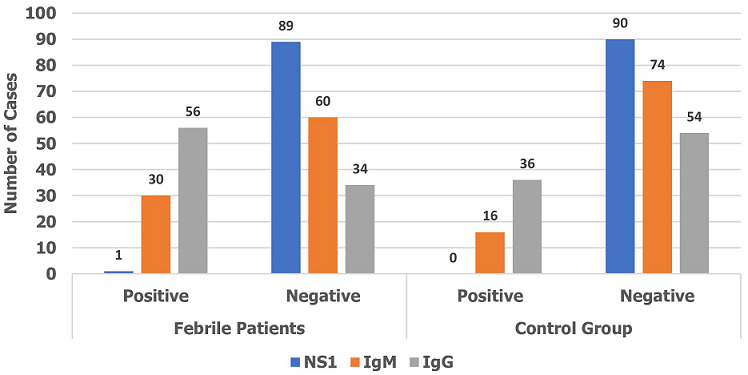
seroprevalence of dengue virus infection among febrile adults and controls

**Seroprevalence of dengue virus infection**: the seroprevalence of recent dengue virus infection (NS1 or IgM positive) was 33.3% (30/90) among the febrile group compared to 17.8% (16/90) in the control group, and the difference was statistically significant (X^2^=5.724, p=0.017). For past DENV exposure (IgG only), the difference in seropositivity was also statistically significantly higher in the febrile group compared to control (62.2% vs. 40.0%, X^2^=8.893, p=0.003). Only 1 (1.1%) febrile participant tested positive for NS1 antigen compared to 0 (0.0%) in the control group. This participant was also Ig M positive. The seroprevalence of dengue virus infection among the febrile participants and controls is shown in Figure 1.

**Clinical presentation of febrile patients according to dengue serological status for recent infection**: only 64/90 (77.1%) had documented fever at presentation. The median (IQR) duration of self-reported fever was 4 (3.0-7.0) days. The most common symptoms reported among the febrile patients were headache (64/90, 71.1%), nausea/vomiting (38/90, 42.2%), muscle pain (38/90, 42.2%) and joint pain (34/90, 37.8%), while pallor (18/90, 20.0%) and abdominal tenderness (8/90, 11.1%) were the most common signs elicited.

When febrile patients were stratified according to those with recent dengue versus those without, there was no statistically significant difference in medical history between those who had recent dengue and those who did not. The most frequent symptoms among those with recent dengue were headache, seen in 22/30 (73.3%), followed by muscle pain in 14/30 (46.7%) and joint pain and nausea/vomiting reported in 12/30 (40.0%) patients each. A history of fever in the last 12 months was reported by 20/30 (73.3%) dengue IgM seropositive febrile patients, which was comparable to 45/60 (75.0%) in dengue IgM seronegative febrile patients (X^2^=0.029, p=0.864). Both groups (dengue IgM seropositive vs. dengue IgM seronegative) were comparable in their physical findings.

**Laboratory parameters of febrile patients according to dengue serological status for recent infection**: as shown in Table 2, neutrophil differential count showed statistically significant difference when dengue IgM seropositive and seronegative febrile adults were compared (X^2^=7.597, p=0.022). The proportion of IgM seropositive patients with haematological abnormalities compared to seronegative were: anaemia (20.0% vs. 20.0%, X^2^=0.000, p=1.000), leucopaenia (16.7% vs. 23.3%, X^2^=1.746, p=0.418) and thrombocytopenia (20.0% vs. 28.3%, X^2^=0.917, p=0.632). The mean liver enzymes, total and conjugated bilirubin and INR were comparable between the two groups. Malaria parasitemia was more frequently seen in the seropositive febrile participants, 23 (76.7%) compared to 33 (55.0%) in the seronegative febrile group; the difference was statistically significant (X^2^=3.994, p=0.046).

**Table 2:** laboratory parameters of participants with and without recent dengue infection

Variable	Dengue IgM Seropositive Febrile Patients N=30 (%)	Dengue IgM Seronegative Febrile Patients N=60 (%)	Test statistics (X^2^)	P-value
**Hb (g/dl)**	-	-	-	-
Mean (SD)	11.7 (1.8)	11.3 (2.1)	-0.777^†^	0.439
< 10	6 (20.0)	12 (20.0)	0.000	1.000
≥10	24 (80.0)	48 (80.0)	-	-
**Total WBC (x10^9^/L)**	-		-	-
Median	5750 (4000-8950)	5050 (3925-7375)	823^w^	0.507
< 4	5 (16.7)	14 (23.3)	1.746	0.418
4-12	23 (76.7)	38 (63.3)	-	-
>12	2 96.7)	-	-	-
**Neutrophils (%)**	-	-	-	-
Median (IQR)	57.5 (52.5-64.5)	55.5 (44-73.8)	862^w^	0.745
<40	0 (0.0)	10 (16.7)	7.597	0.022*
40-75	27 (70.0)	39 (65.0)	-	-
>75	3 (23.3)	11 (18.3)	-	-
**Lymphocyte (%)**	-	-	-	-
Median (IQR)	38 (32.0-45.0)	41 (23.3-50.8)	863^w^	0.755
<20	0 (6.7)	10 (16.7)	4.603	0.100
20-45	21 (70.0)	28 (46.7)	-	-
>45	7 (23.3)	22 (36.7)	-	-
**Platelet (x10^9^/L)**	-		-	-
Median	208 (166.5-266.8)	224 (143.5-293.5)	841^w^	0.611
<150	6 (20.0)	17 (28.3)	0.917	0.632
150-450	23 (76.7)	42 (70.0)	-	-
> 450	1 (3.3)	1 (1.7)	-	-
**ALT (IU/L)**, Mean (SD)	9.8 (1.8)	9.9 (3.7)	0.188^†^	0.851
**AST (IU/L)**, Mean (SD)	9.2 (2.0)	9.4 (3.9)	0.326^†^	0.745
**ALP (IU/L)**, Mean (SD)	63.2 (18.7)	56.8 (19.3)	-1.490^†^	0.140
**Total bilirubin** (µmol/l), Mean (SD)	11.5 (3.3)	10.4 (3.8)	-1.385	0.170
**Conjugated bilirubin** (µmoles/l), Median (IQR)	3.4 (1.7-5.1)	3.4 (1.7-3.4)	839^w^	0.586
**INR**, Mean (SD)	1.0 (0.1)	1.0 (0.1)	-	-
**Malaria parasitemia**	-	-	-	-
Yes	23 (76.7)	33 (55.0)	3.994	0.046*
No	7 (3.3)	27 (45.0)	-	

* Statistically significant at p<0.05, ^†^- Student's T test, SD - standard deviation, X^2^ - Pearson Chi square, W - Mann Whitney U, Hb= Haemoglobin, WBC= White blood cell count, ALT= Alanine transaminase, AST= Aspartate transaminase, ALP= Alkaline phosphatase INR= International normalized ratio

**Clinical interpretation of dengue serological results in consideration of onset of fever and malaria parasitaemia**: as shown in Table 3, none of the febrile participants met the clinical criteria for definitive dengue fever (subject to a polymerase chain reaction, PCR). However, 7/90 (7.7%) persons had findings highly suggestive of dengue fever. Of these, 1 (1.1%) person who reported fever after 7 days of its onset tested positive for NS1 antigen and had anti-dengue IgM antibodies without malaria parasitemia. The other 6 (6.7%) comprised those who presented after 7 days of onset of fever and had anti-dengue IgM antibodies but no NS1 antigenemia. Of these 6, 2 had no malaria parasitemia, while 4 had malaria parasitemia. Probable dengue fever was considered in 23/90 (25.6%), with 19 (33.9%) positive for malaria parasitemia, while 35 (38.9%) patients were classified as having solitary anti-dengue IgG antibodies. The remaining 25 (27.8%) patients comprised those who tested negative for all dengue serological markers and were classified as having no dengue infection. In general, dengue seropositive participants had detectable malaria parasitemia more frequently compared to dengue seronegative participants (61/107; 71.8% vs 24/73; 28.2%, p=0.001), while DENV-Malaria coinfection was seen in 44/90 (48.9%) febrile patients.

**Table 3:** clinical interpretation of dengue serologic results in consideration of the onset of fever and stratified by malaria parasitemia status

Patient Classification (Clinical interpretation)	Dengue serological finding	Febrile patients N=90 n (%)	Positive Malaria Parasite N=56 n (%)	Negative Malaria Parasite N=34 n (%)
Definitive dengue fever (subject to PCR)	IgM positive and NS1 positive within 7 days of the onset of fever	0 (0.0)	0 (0.0)	0 (0.0)
Highly suggestive of dengue fever	IgM negative and NS1 positive within 7 days of the onset of fever	0 (0.0)	0 (0.0)	0 (0.0)
	IgM positive and NS1 positive after 7 days of the onset of fever	1 (1.1)	0 (0.0)	1 (2.9)
	IgM positive and NS1 negative after 7 days of the onset of fever	6 (6.7)	4 (7.1)	2 (5.9)
Probable dengue fever	IgM positive and NS1 negative within 7 days of onset of fever	23 (25.6)	19 (33.9)	4 (11.8)
	IgM negative and NS1 positive after 7 days of the onset of fever	0 (0.0)	0 (0.0)	0 (0.0)
Anti-dengue IgG seropositive	IgG positive only, irrespective of the duration of fever	35 (38.9)	21 (37.5)	14 (41.2)
No dengue infection	Serologic markers negative for all; IgG, IgM, and NS1	25 (27.8)	12 (21.4)	13 (38.2)

NS1-Non-structural protein 1, IgM- Anti-dengue immunoglobulin M, IgG- Anti-dengue immunoglobulin G, PCR-Polymerase chain reaction

**Risk factors associated with recent dengue infection among febrile adults**: of the variables subjected to bivariate analysis (Table 1), age 40 years and below (83.3% vs. 16.7%, p=0.051), malaria parasitemia (76.7% vs. 23.3%, p=0.046) and self- report of having window/door nets (86.7% vs. 13.3%, p=0.083) were marginally associated with recent dengue infection. On multivariate analysis that included age, those who had window or door nets, those who reported having bushes/pools of water around their home, diabetes mellitus and malaria parasitemia (all with p < 0.25), having window/door nets (AOR= 3.67, 95% CI 1.02 to 8.21, p=0.045) was an independent predictor of recent dengue virus infection. Additionally, the odds of recent dengue infection were observed to be 2.5 times higher in those who reported having bushes/pools of water around their home, while persons who had malaria parasitemia had 3.0 times the odds of having serological evidence of recent dengue infection.

**Risk factors associated with past dengue infection among febrile adults**: for past dengue, male sex compared to female (54.3% vs. 45.7%, p=0.087), self-report of having window/door nets (62.9% vs. 37.1%, p=0.153) and hypertension (42.9% vs. 57.1%, p=0.064) were marginally associated with past dengue infection on Chi-square analysis. None of the participants had a history of travel to an endemic region in the last month, nor had any received an organ transplant. Only one febrile participant received a blood transfusion in the preceding month; likewise, only one had contact with a person with suspected VHF.

After controlling for cofounders on multivariate analysis using factors with a p-value <0.25 on Chi-square analysis, no variable predicted past DENV infection. However, males had 2.4 times the odds of having evidence of past dengue infection, while participants who reported window or door nets had lower odds of dengue infection (OR = 0.42; 95% CI: 0.12-1.46; p = 0.170) (Table 2).

## Discussion

This study found that the seroprevalence of recent (IgM, or NS1) and past DENV (IgG) infection was significantly higher in febrile participants compared to healthy controls, while having window/door nets was identified as an independent predictor of recent dengue infection.

**Dengue seroprevalence**: serological evidence of recent dengue infection (IgM seropositivity) was found in one-third of the febrile patients in our study, which is similar to the 37.4% reported by Hamisu *et al*. in Borno [32]. The seroprevalence of anti-dengue IgM in previous studies in Nigeria ranges from 23.3% to 51.6% [15,21,33,34]. These discrepancies may be due to geographical variations, climatic differences, and population dynamics. NS1 antigenemia, a reliable marker for early dengue infection, was found in only one of the febrile patients in this study. This finding is similar to 2.2% found in Jos [35] and 2.8% reported in Uyo [20]. Contrarily, Hamisu *et al*. reported 9.9% in Borno [32] while NS1 antigenemia was absent in a Malaysian population studied [13]. The consistently low levels of NS1 antigenemia may be related to periodicity, as NS1 appears early in the infection process and is short-lived. It is therefore most useful among persons presenting early to the health facility.

A higher seroprevalence of anti-DENV IgG (62.2%) was seen among the febrile patients in this study compared to another study that involved HIV-infected febrile patients in Abuja, which reported anti-DENV IgG of 44.4% [36]. A previous study in India [37] had reported anti-DENV IgG of 74.7% in febrile subjects, while other household surveys done in various parts of the country have reported anti-DENV IgG ranging from 42.6% to 67.2% [12,38]. These studies, including the finding of anti-DENV IgG in nearly half of this study's controls collaborate the endemicity of DENV in Nigeria.

**Clinical characteristics and interpretation of dengue seropositivity in febrile patients**: symptoms frequently reported in dengue IgM seropositive febrile patients in this study were headache (73.3%), muscle pain (46.7%), joint pain and nausea/vomiting (all in 40.0%). These symptoms, although frequently encountered, were non-specific as none showed statistical significance when dengue IgM-positive versus IgM-negative febrile patients were compared. Despite the self-report of fever in all within the febrile group, only in three-quarters of them did we document fever by axillary temperature recording. The high frequency of over-the-counter use of antipyretics may have contributed to our temperature findings. Also, the presence of intermittent fever patterns, which is most often subjectively reported, could have played a role. Three-quarters of the febrile adults in this study reported suffering at least one previous febrile illness in the preceding 12 months. While this study was not focused on unravelling evidence of co-infection by a wide spectrum of pathogens, this observation lends credence to the need for incorporating assessment for dengue in the evaluation of undifferentiated fever in our environment and improving diagnostic assays towards unravelling the aetiology. This study found that IgM seropositive febrile participants did not differ from seronegative febrile participants in the laboratory markers assayed. Nevertheless, FBC and LFT are useful investigations in the evaluation of febrile patients as progressive decline in total WBC is one of the earliest abnormalities in certain infections such as dengue [39]. Thrombocytopenia is also an early pointer towards dengue infection and has been implicated in severe dengue presentations [40]. In dengue, ALT and AST are usually increased and may suggest the severe dengue forms [41,42].

Despite the absence of confirmatory PCR, serological markers in conjunction with the duration of the fever have been shown to provide good clinical guidance for the provisional diagnosis of dengue fever [29]. No participant in this study met the criteria for a definitive case of dengue fever. However, less than one-tenth of the febrile adults had their presentation and serological markers highly suggestive of dengue fever, and another one-quarter of febrile adults met the criteria for probable dengue fever. Both groups documented co-existent malaria parasitemia seen in about 7% of those categorised as highly suggestive of dengue and in one-third of those classified as probable dengue fever. The much lower rates of malaria parasitemia in the greater majority of those with findings highly suggestive of dengue fever may be a pointer to the role of arboviral infections such as dengue and maybe other non-malarial pathogens in the aetiology of acute febrile illness in these patients. This corroborates the finding of an inverse relationship between the occurrence of dengue and malaria in India [16]. Those categorised as anti-dengue IgG seropositivity comprised two-fifths of the febrile adults. Although the relative proportion of each of the clinical interpretations of dengue diagnosis (definitive dengue, highly suggestive of dengue, probable dengue, anti-dengue IgG) in the present study is similar to the relative proportions reported in a previous study in Nigeria (2.3%, 5.5%, 1.5%, and 11.7%, respectively), the absolute percentages of patients diagnosed with features highly suggestive of dengue, probable dengue, and anti-dengue IgG seropositivity were much higher in the present study [29]. This is possibly due to our relatively smaller sample size, which comprised only adults, while that in Jos involved more participants and comprised both adults and children [29]. Additionally, geographical differences in vector population and transmission of infection may have played a role.

**Risk factors associated with recent dengue infection**: this study identified only those who reported having a window/door net as an independent risk factor for recent dengue infection. These persons had higher odds of DENV exposure. This differs from Eldigail *et al*. finding that non-adoption of mosquito control practices was a predictor for dengue seropositivity [11]. Our findings should be interpreted with caution as it is expected that the impact of having a window/door net will be better utilised in accessing long term effect rather than the recent effect, which IgM addresses. How long participants had the reported nets, or the integrity of these nets, is called into question. It is also possible that this is a protective adaptive behaviour in the face of a high burden of mosquitoes around the living quarters.

Of the variables accessed, none was an independent predictor for evidence of past dengue infection in this study. This finding is somewhat similar to that in Pakistan, where there was no association between IgG seropositivity and selected variables, but differs from some earlier reports where sex and age, amongst others, were predictors of past exposure to DENV [10,13,43]. The presence of window/door nets in homes was protective from past dengue infection, suggesting that consistent use of these environmental and mosquito control measures may be useful adjuncts in the control of DENV infection, though these benefits may be realised in the long term. This is consistent with vector control practices being reported and advocated as vital in the prevention and control of dengue [1].

**Limitation:** Despite the high specificity of the DENV ELISA kits, cross-reactivity of other flaviviruses, especially the yellow fever virus and the limited spectrum of the pathogens interrogated remain a potential limitation of this study and should be taken into consideration while interpreting the findings. In Nigeria, possible cross-reaction with the yellow fever virus is of particular interest, given the outbreaks of yellow fever in various States of the country, especially in the past few years [44]. The study design did not permit assay of a convalescent sample; hence, caution should be applied in interpreting the diagnostic relevance of a single-sample serological assay. Additionally, although we preferentially targeted enrolment of eligible relatives of cases as controls, due to the limited number of available/eligible relatives, eligible hospital staff were also recruited as controls. We recognise that such hospital staff, although resident in the study's targeted geographical region, may have other epidemiologic characteristics that we may not fully account for. Also, the drawbacks of a single-centred study threaten the generalisability of findings, but do not negate the importance of these findings.

## Conclusion

This study found a relatively high seroprevalence of recent DENV, with prevalence higher in febrile patients compared to apparently healthy controls. High malaria burden with dengue-malaria co-infection was frequently observed, while those who reported having a window/door net were independent predictors of recent dengue infection. There is a need to improve mosquito control practices while incorporating routine laboratory diagnostics for dengue in evaluating febrile persons in SSA, irrespective of the presence of malaria parasitemia. Further studies utilising PCR to confirm the presence of DENV infection and identify the implicated serotype(s) are recommended.

### What is known about this topic

- Dengue virus is a neglected tropical disease and the most common mosquito-borne virus, it has been previously isolated in patients in Nigeria and sub-Saharan Africa;
- Dengue may lead to severe presentations that are potentially life-threatening, especially with delayed recognition and diagnosis.

### What this study adds

- We found a relatively high prevalence of recent dengue (33.3%) among febrile patients attending a Nigerian tertiary hospital;
- It collaborates with existing evidence that non-specific laboratory tests, such as haematological and biochemistry analytes, are of little value in establishing a diagnosis of dengue.
